# Human area MT^+^ shows load-dependent activation during working memory maintenance with continuously morphing stimulation

**DOI:** 10.1186/1471-2202-15-85

**Published:** 2014-07-11

**Authors:** Daniela Galashan, Thorsten Fehr, Andreas K Kreiter, Manfred Herrmann

**Affiliations:** 1 Department of Neuropsychology and Behavioral Neurobiology, Center for Cognitive Sciences (ZKW), University of Bremen – Cognium Building, Hochschulring 18, D-28359 Bremen, Germany; 2 Center for Advanced Imaging (CAI), University of Bremen, Bremen, Germany; 3 Institute of Brain Research, University of Bremen, Bremen, Germany; 4 Department of Neurology, Otto-von-Guericke University, Magdeburg, Germany

**Keywords:** fMRI, Human, hMT, V5, Retention

## Abstract

**Background:**

Initially, human area MT^+^ was considered a visual area solely processing motion information but further research has shown that it is also involved in various different cognitive operations, such as working memory tasks requiring motion-related information to be maintained or cognitive tasks with implied or expected motion.

In the present fMRI study in humans, we focused on MT^+^ modulation during working memory maintenance using a dynamic shape-tracking working memory task with no motion-related working memory content. Working memory load was systematically varied using complex and simple stimulus material and parametrically increasing retention periods. Activation patterns for the difference between retention of complex and simple memorized stimuli were examined in order to preclude that the reported effects are caused by differences in retrieval.

**Results:**

Conjunction analysis over all delay durations for the maintenance of complex versus simple stimuli demonstrated a wide-spread activation pattern. Percent signal change (PSC) in area MT^+^ revealed a pattern with higher values for the maintenance of complex shapes compared to the retention of a simple circle and with higher values for increasing delay durations.

**Conclusions:**

The present data extend previous knowledge by demonstrating that visual area MT^+^ presents a brain activity pattern usually found in brain regions that are actively involved in working memory maintenance.

## Background

### Area MT^+^ and memory

The term ‘MT^+^’ is used when referring to a brain area comprising the putative human homolog of MT proper medial temporal area; [1], and medial superior temporal cortex (MST). Human MT^+^ complex was primarily considered a purely motion sensitive region [2], but later studies have identified subregions showing object-selective characteristics [3,4]. Moreover, MT^+^ seems to be involved in the processing of various motion-related processing mechanisms like mental rotation [5], imagery of motion [6], implied motion [7], or linguistically derived motion expectation [8]. Additionally, several authors pointed to an involvement of area MT^+^ in memory processing [9,10]. For example, memory for motion direction was associated with increased neuronal activity in MT during retention [10] and human MT^+^ was shown to demonstrate a signal increase in functional magnetic resonance imaging (fMRI) when motion information had to be held in working memory (WM) [11].

These memory studies investigated motion-related memory contents. Here, we investigated whether area MT^+^ is also engaged during WM maintenance when no motion-related WM content is used, but motion information has to be processed.

### Persistent maintenance activity

Previous research mainly focused on brain activity in various other brain areas during WM maintenance. Single cell studies in macaque monkeys, for instance, identified neuronal activity during WM maintenance in frontal [12], posterior parietal [13], and (inferior) temporal cortices [14]. An important finding concerning this delay spanning activity is the fact that it persists in prefrontal cortex when distractor stimuli are presented during retention, but is disrupted in inferior temporal [15] and posterior parietal cortex [16,17]. This persistent activity was suggested to indicate active maintenance of the stimulus’ representation [18], even if the proper representation is held somewhere else in the brain [19]. Thus, in the present study, we used continuously changing (morphing) and therefore distracting stimuli during the maintenance period to provoke active WM maintenance and more focused task processing.

### Working memory load

In addition to persistent maintenance activity another characteristic of WM retention is the modulation by WM load, e.g. [20]. An increase in memory load leads to more elaborate WM task processing and is commonly provoked by enlarging the number of items (item set size, e.g., [21]) increasing the complexity of items [22], or extending the duration of the retention period [23]. In the present study we aimed at both, i.e. modulating WM load by the complexity of the memorized target shapes (complex shapes, simple circle) and parametrically increasing the delay duration (3/6/9/12 s). Given this experimental procedure, our design was assumed to induce active WM maintenance for complex compared to simple shapes. We explored whether manipulations in complexity and delay length modulate brain activity in area MT^+^ during WM maintenance, and whether area MT^+^ is specifically engaged in the processing of stimuli with motion information that does not need to be maintained in WM.

In summary, the data show higher PSC (percent signal change) values in area MT^+^ during maintenance of complex compared to simple shapes as well as higher values with increasing delay duration. This type of activity pattern with higher activation during the retention period despite distracting stimulation has usually been associated with brain regions actively involved in WM maintenance.

## Methods

### Study participants

Nineteen healthy students (9 male; 20–30 years; mean age = 24.8; SD = 3.0) participated in the experiment after giving informed and written consent. They received 10 € for their participation. Four data sets had to be excluded due to technical problems, misunderstanding of the instructions, or severe motion artefacts in the fMRI data set. All participants were right-handed according to the modified Edinburgh Handedness Inventory [24] and had normal or corrected-to normal vision. Participants did not report a history of neurological and/or psychiatric disorders, or medication or substance abuse affecting the central nervous system. The study protocol was conducted according to the Helsinki Declaration of the World Medical Association [25] and approved by the Ethics Committee of the University of Bremen.

### Stimuli

**Figure 1:**
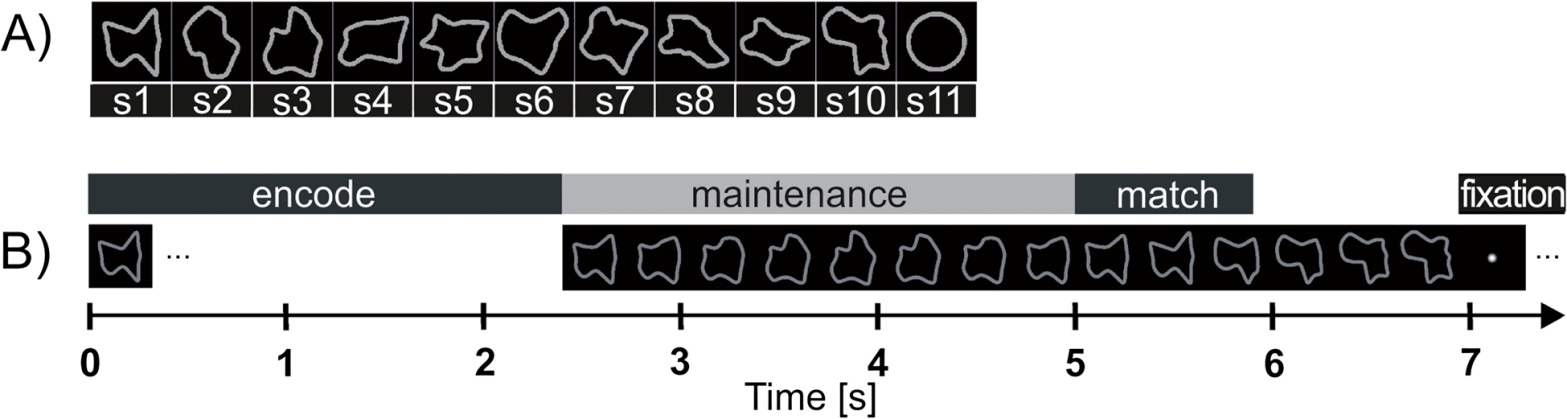
**Stimuli and trial sequence. A)** Shapes used for the morphing delayed match-to-sample task. Complex shapes: (S) 1 to 10; simple circle shape (S) 11. **B)** Trial sequence: A static target was presented for 2.4 s and subsequently started to continuously change its contours and morphed with a smooth motion into another of the predefined shapes. The initial target shape reappeared after 3, 6, 9 or 12 s, and the participants had to indicate the recognition of the target shape by a button press. After complete presentation of the target shape, the sequence morphed into one other shape in order to prevent trial termination with the target.

Stimuli consisted of complex curved shapes that could not be easily identified through salient features or verbalization and a simple circle as a low-level control condition. A set of eleven different shapes consisting of gray outlines covering 3.3-4.9° angle of vision was used (Figure 1A). These shapes were defined by 16 (non-visible) points connected by a smooth Bezier curve. For the control condition (circle) these points were distributed on a unit circle with a constant angle. Complex shapes were generated by jittering the angle (σ = 0.7°) and the radius (0.3° visual angle) of each point following a normal distribution. Morphing between two subsequent shapes was realized by moving the points of one shape on a linear trajectory to the position of the points of the following shape within 1.5 s [26].

### Task presentation

The trial sequence is shown in Figure 1B. After an encoding epoch (2.4 s) with a static presentation of the target shape, the shape continuously morphed into the other probe shapes. During this morphing process a new probe stimulus from a predefined set of ten shapes was presented every 1.5 s. Participants were instructed to press a button with the right index finger as soon as they recognized the target shape. The maintenance period (i.e., the time between encoding epoch and complete reappearance of the target) lasted either for 3, 6, 9 or 12 seconds, corresponding to a sequence of 1, 3, 5 or 7 non-matching probe configurations, respectively. After full reappearance of the target stimulus the morphing sequence continued for another 1.5 seconds to avoid a predictable stimulation pattern with all trial sequences terminating with the target stimulus. Trials were separated by jittered inter-trial intervals (2 ± 0.3 s) where only a fixation point was presented.

In the control condition a circle was used as target. The circle never appeared during the maintenance period, and only the complex shapes were used as morphing non-targets during this time window. There was no repetition of a previous shape during the morphing process within a trial.

By presenting a limited set of constantly reccurring stimuli as complex sample and probe stimuli, participants were not able to rely on judgments based on familiarity. They had to actively maintain the current shape in WM in order to recognize it. All complex shapes were used equally distributed as targets within each delay period. Furthermore, each complex shape was presented with the same frequency as first probe stimulus after the encoding epoch and as last probe stimulus before target-reappearance in order to prevent trial position effects.

Stimuli were presented in four separate runs in a counterbalanced order. Each run consisted of 30 complex trials and 20 control trials that were presented in a pseudo-randomized order, and the frequency of trial type within each delay duration period was kept constant across all runs. All participants performed a short practice run outside the scanner (4 complex and 2 control trials). The stimulation procedure was realized with Presentation®-Software 9.90 (Neurobehavioral Systems, Albany, CA).

### Behavioral data analysis

Only trials with a correct response within a time window of 400 ms before to 500 ms after full reappearance of the target shape were used for the analysis of reaction times (RTs). Error trials (false positives and omissions) were excluded from RT analysis and analyzed separately. RT data were subjected to a repeated-measures three-way analysis of variance with target complexity (complex, simple) and delay duration (3 s, 6 s, 9 s, 12 s) as within-subjects factors and run order (1,2,3,4 or 4,3,2,1) as between-subjects factor. Percent error rates were analyzed with Friedman tests separately for complex and for control trials with the four delay durations as different variables. Post hoc comparisons between the error rates of the different delay durations and between simple and complex trials were examined with Wilcoxon tests for paired samples.

### MRI data acquisition and fMRI data analysis

MRI data were obtained using a 3 T Allegra® scanner (Siemens, Erlangen, Germany) equipped with a standard quadrature head coil. Participants lay on a scanner couch in a dimly lit scanner room and wore foam earplugs. Functional images were acquired using an echo-planar imaging (EPI) sequence (TR = 2.06 s, TE = 30 ms, flip angle = 80°, 64 × 64 matrix, FOV 192 × 192 mm, 3 mm^3^ voxels, interleaved slice acquisition, 38 slices). WM task runs (12 min each, separated by 1–5 min breaks) consisted of 343 volumes. To identify functionally defined motion-sensitive area MT^+^, a localizer scan was accomplished with 202 volumes (see below).

For T1-weighted anatomical images an MPRAGE sequence was used (TR = 2.3 s, TE = 4.38 ms, flip angle = 8°, TI = 900 ms, FOV 256 × 256, 1 mm^3^ voxels, 160 slices, duration: about 10 min).

Pre-processing and first-level analyses of the functional MR data was conducted with SPM2 (Wellcome Trust Centre for Neuroimaging, London, UK), all other data analyses (localizer task and 2^nd^-level analysis of the WM data) were carried out with SPM5. Pre-processing of the WM data included motion correction (realigned to the 10^th^ volume of each run determining six parameter rigid-body transformations; realign and unwarp algorithm), slice timing correction, and normalization to the MNI standard space including resampling to isotropic voxels of 2 mm^3^. Functional data were spatially smoothed applying a 6 mm full width at half maximum (FWHM) Gaussian kernel, serial autocorrelations were corrected using a pre-whitening procedure (AR(1) correction), and low frequency drifts were removed with a high-pass filter of 128 s.

For the analysis of the WM task functional data, encoding and retrieval periods were modeled separately for complex and simple control trials as a boxcar function convolved with a canonical hemodynamic response function [27,28], whereas duration of the delay period was modeled independently. Maintenance duration was defined as the time interval starting with the morphing process and terminating when the pre-target shape started morphing into the target shape in order to ensure that retrieval processes were modeled separately from the maintenance condition. Trials with errors and misses were combined separately per delay duration and were defined as regressors of no interest. The six realignment parameters (x, y, z translation, and three rotation angles) were included as regressors of no interest in order to handle movement-related artefacts [29].

Voxel-wise fixed-effects contrasts were performed on single subject level and the resulting individual contrasts were subjected to a multi-subject random effects analysis [30]. Maintenance periods for the complex trials were contrasted with those for the simple control trials separately for each delay period. We introduced a conjunction (null) analysis [31] to investigate combined activation patterns across all delay durations (complex vs. simple for 3, 6, 9 and 12 s delay) because it ensures that the resulting regions were activated in each individual delay condition. The statistical threshold for contrasting procedures was fixed at a significance level of p ≤ .001 with FDR correction [32,33]. Significant clusters had to consist of more than 20 contiguous voxels to be considered for further analysis (k > 20). Peak coordinates of regions activated during the WM task were converted from MNI to Talairach space using the Brett transformation ‘Mni2tal’ [34].

### MT^+^ localizer scan and regions of interest analysis

The MT^+^ localizer consisted of three conditions: (1) A static field with 500 gray squares of variable size on a black background, (2) a moving flowfield where the gray squares moved radially, alternating between expansion and contraction movements with an observer speed of 150 cm/s, and (3) a resting condition with a blank screen. All three conditions contained a red fixation point (12 arcmin) at the center of the screen and participants were instructed to maintain fixation on this point during the whole MT^+^ localizer scan (6 blocks of conditions 1 and 2, lasting 20.6 s each; with 12.4 s blocks of condition 3 interleaved).

EPI volumes from the MT^+^ localizer runs were pre-processed as described before except for a co-registration of the individual T1 images to the 10^th^ volume of the functional data and a segmentation of the individual T1 image into cerebrospinal fluid, gray and white matter. The resulting segmentation parameter file was used to normalize the functional data, which were subsequently smoothed with an 8 mm FWHM Gaussian filter.

Area MT^+^ was determined as the region showing a significantly higher signal increase in the motion condition compared to the static condition on a single subject level. The resulting Talairach coordinates of area MT^+^ averaged over each individual MT^+^ center of mass (mean ± SD; right: 49 ± 2.6; −64 ± 3.6; 4 ± 3.4/left: −46 ± 4.4; −69 ± 4.6; 7 ± 4.2) were in line with MT^+^ coordinates reported in other studies [3,35,36].

PSC values were calculated separately for all delay conditions of complex and simple trials and for the retrieval periods on smoothed data using the Marsbar toolbox [37] and averaged over both hemispheres for each region of interest. PSC data are reported from 12 participants only who showed bilateral MT^+^ activation. For visualization purposes all individually localized regions of interest were overlaid using the Marsbar toolbox.

## Results

### Behavioral data

Response times were calculated starting with the time point when the target completely reappeared.

**Figure 2:**
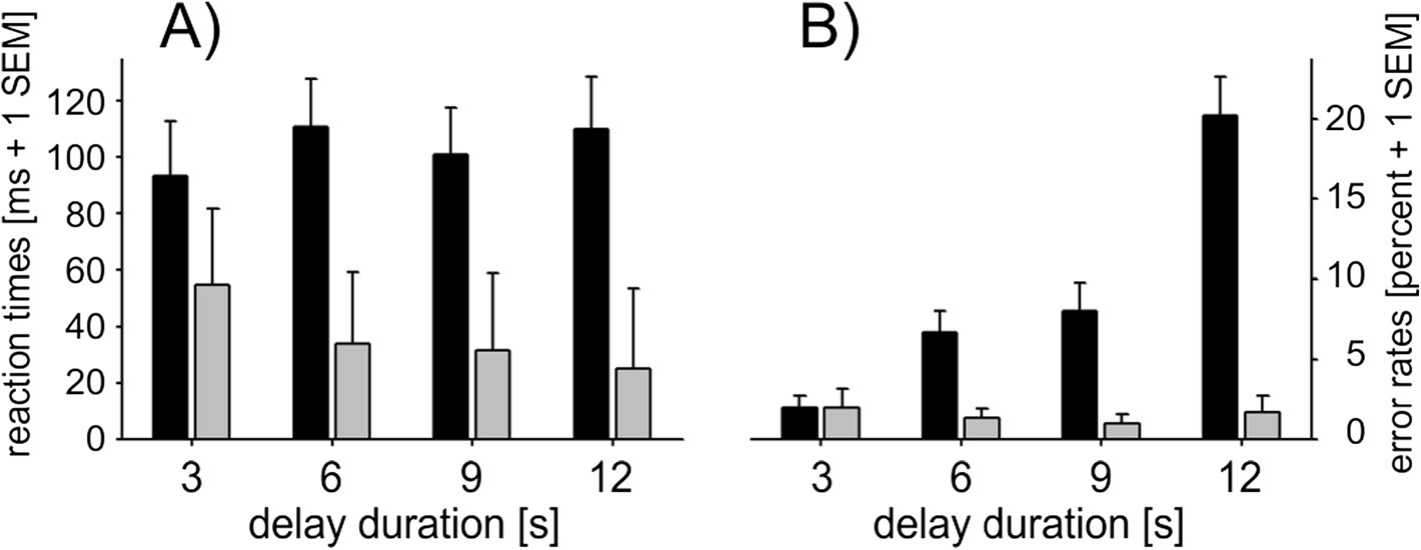
**Behavioral data.** Reaction times **(A)** and percent error rates without misses **(B)** for complex trials (black) and simple control trials (gray) separately for all delay durations. Error bars represent 1 SEM.

A three-way ANOVA of RT data including target complexity (complex, simple), delay duration (3, 6, 9, 12 s) and run order (regular/inverse) showed a significant main effect of target complexity (F_[1,13]_ = 22.5; p < .001) indicating faster responses to the simple circle control (circle: mean ± S.E.M: 37.2 ± 30.7) compared to complex shapes (mean ± S.E.M: 102.9 ± 19.1 ms; see Figure 2A).Wilcoxon tests comparing error rates of simple and complex trials separately for each delay period resulted in higher error rates for complex shapes for all but the three seconds delay period (3 s: z = −0.1, p = .94; 6 s: z = −2.8, p = .005; 9 s: z = −2.7, p = .007; 12 s: z = −3.4, p = .001; see Figure 2B).

Friedman tests showed significant differences between the four delay conditions for complex trials (χ^2^_[df = 3]_ = 23.9, p < .001) but no significant effects for simple trials (χ^2^_[df = 3]_ = 0.3, p = .97). Post hoc Wilcoxon tests revealed higher error rates with increasing delay duration for complex trials except for the 9 seconds versus 6 seconds delay (6 s vs. 3 s: z = −2.4, p = .02; 9 s vs. 3 s: z = −2.4, p = .02; 12 s vs. 3 s: z = −3.4, p = .001; 9 s vs. 6 s: z = −0.5, p = 0.65; 12 s vs. 6 s: z = −3.2, p = .002; 12 s vs. 9 s: z = −3, p = .003). The number of omissions did not significantly differ between delay durations.

### Activation patterns over different delay periods

**Figure 3:**
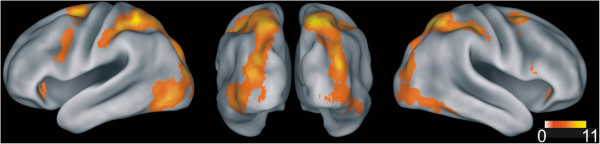
**fMRI activation during maintenance.** Rendered brain statistics superimposing activation of the conjunction over all delay durations (utilizing the 4 contrasts ‘maintenance of complex trials vs. maintenance of simple trials’ of each delay duration; FDR-correction; p < .001, k > 20). Left: left hemisphere, middle: back view of both hemispheres, right: right hemisphere. The bar indicates color assignments of t-values.

We did not find any supra-threshold clusters (FDR-correction; p < .001, k > 20) when comparing the contrast ‘complex > simple maintenance’ between different maintenance conditions. Conjunction analysis over all delay conditions (conjunction null: Complex > simple maintenance for each delay duration contrast) revealed two widespread activation clusters extending bilaterally from inferior and middle occipital gyri to middle and inferior temporal gyri and spreading to inferior and superior parietal areas with two activation peaks in the right hemisphere in superior parietal lobule and one sub-gyral peak in the vicinity of the inferior parietal lobule and in the left hemispheric inferior parietal sulcus, precuneus and middle occipital gyrus (see Figure 3 and Table 1 for the corresponding Talairach coordinates). Further suprathreshold clusters were located in bilateral middle frontal gyri. The left-hemispheric middle frontal cluster showed one peak activation in middle frontal gyrus and two in inferior frontal gyrus. In the right hemisphere, the middle frontal activations consisted of two smaller clusters in middle frontal gyrus (see Table 1).

**Table 1:** Fronto-parietal network consistently involved in WM maintenance under higher memory load conditions

**Anatomical region (BA, r)**	**Side**	**Cluster size (voxels)**	**t-value**	**Peak coordinate**		
				**x**	**y**	**z**
Superior parietal lobule (BA 7, r = 5)	R	4899	11.59	18	−65	55
Superior parietal lobule (BA 7, r = 3)			9.75	26	−58	53
Inferior parietal lobule (BA 40, r = 5)			9.72	40	−33	46
Inferior parietal lobule (BA 40)	L	5585	9.70	−36	−44	48
Precuneus (BA 7, r = 3)			9.22	−18	−69	51
Middle occipital gyrus (BA19)			9.21	−46	−74	−5
Middle frontal gyrus (BA 6, r = 3)	L	1146	9.69	−28	−1	55
Inferior frontal gyrus (BA 9)			6.05	−57	9	27
Inferior frontal gyrus (BA 9, r = 5)			6.02	−50	9	24
Middle frontal gyrus (BA 6, r = 3)	R	827	9.61	30	1	57
Inferior frontal gyrus (BA 47, r = 5)	L	155	6.56	−32	21	−3
Medial frontal gyrus (BA 6, r = 3 /BA32)	L	330	6.47	−4	14	47
Medial frontal gyrus (BA 6)			6.19	6	16	45
Inferior frontal gyrus (BA 47, r = 7)	R	109	6.36	32	25	−5
Lentiform nucleus	L	39	5.29	−12	2	0
Middle frontal gyrus (BA 8, r = 3)	R	54	4.93	48	8	42
Middle frontal gyrus (BA 6, r = 3)			4.78	42	4	38
Cerebellum culmen	R	22	4.92	36	−53	−21
Caudate head (r = 3)	R	33	4.87	12	8	1
Inferior frontal gyrus (BA 44, r = 5)	R	42	4.77	50	11	22
Inferior frontal gyrus (BA 44, r = 3)			4.40	51	12	12
Inferior frontal gyrus (BA 45, r = 5)			4.24	57	15	21
Cerebellum declive	R	49	4.67	28	−75	−16

Talairach coordinates of activation peaks revealed by a conjunction analysis of the maintenance period of complex trials versus maintenance period of simple trials conjunct over all four delay durations (FDR-corrected, p < .001; k > 20). Anatomical labels of peak activations are listed in boldface and significant sub-peaks are listed without boldface. Abbreviations: BA = Brodmann area; r = range of nearest gray matter in mm; R = right hemisphere; L = left hemisphere.

### Modulation of area MT^+^ (percent signal changes, PSC)

**Figure 4:**
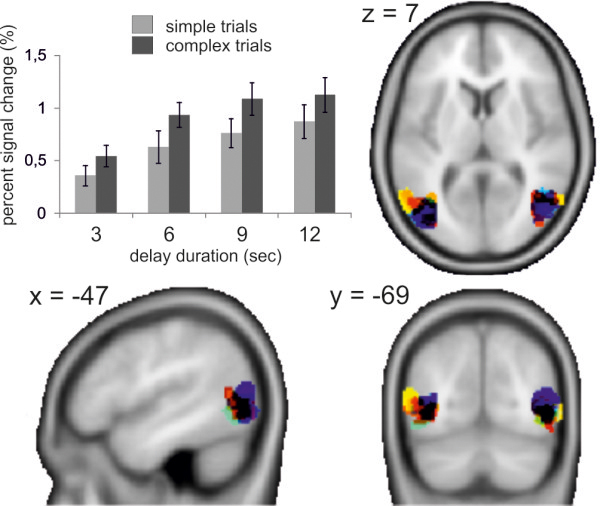
**Percent signal change in area MT**^**+**^**.** Bar chart for percent signal change in MT^+^ (left) for the maintenance period of simple trials (light gray) and complex trials (dark gray), separately for delay durations of 3, 6, 9, and 12 seconds. The axial, sagittal and frontal brain slices demonstrate overlays of MT + ROIs derived from individual localizer scans (N = 12).

PSC values from area MT^+^ were averaged over both hemispheres as Wilcoxon tests did not show significant differences between the corresponding values. Wilcoxon tests for paired samples yielded significantly higher PSC values for complex compared to simple maintenance epochs for each delay condition (3 s: p = .012; 6 s: p = .001; 9 s: p = .002; 12 s: p = .002) in MT^+^ (see Figure 4). This effect was remarkably consistent over all participants: There were only three participants showing higher PSC values for simple compared to complex trials in at least one condition (one participant for left area MT^+^ in the 3 s and 12 s condition and also for right area MT^+^ in the 3 s condition; two other participants each in the 3 s condition in left area MT^+^).

Friedman tests for PSC data from area MT^+^ revealed significant differences between the four delay durations for complex trials (χ^2^_[df = 3]_ = 29.5, p < .001) as well as for simple trials (χ^2^_[df = 3]_ = 24.1, p < .001). Post-hoc Wilcoxon tests statistically confirmed increasing PSC values with increasing delay duration for complex as well as for simple trials when comparing delay durations except for the comparison of the 9 s versus 12 s delay duration of complex trials (complex trials: z ≥ −2.7, p ≤ .008 for all comparisons except 9 s vs. 12 s/simple trials: z ≥ −2.5, p ≤ .015 for all comparisons).

Separate Wilcoxon tests of MT^+^ PSC values during the retrieval period for each delay condition did not show any significant differences between complex and simple trials for any of the four delay durations.

## Discussion

### Summary of results

We used a WM paradigm with a continuous stimulation by morphing shapes during the maintenance epoch. WM load was systematically modulated by the complexity of the memorized target stimulus and by delay duration. A simple circle condition was chosen as a low-level baseline with identical visual stimulation properties, task instructions and demands on motor processing. Thus, the only difference between the contrasted maintenance periods for complex versus simple shapes was the complexity of the memorized stimulus material.

Maintenance of complex as compared to simple stimuli engaged a large network of brain areas that was involved consistently during all delay durations. Furthermore, activity in motion-sensitive area MT + demonstrated a brain activation pattern with higher PSC values when complex shapes were memorized, and increasing values with prolonged delay duration.

### Task performance

Behavioral data analyses showed better task performance for simple as compared to complex targets. Shorter RTs in simple trials might simply indicate an earlier identification of the circle compared to the complex shapes during the morphing period. In addition, lower error rates for simple trials might reflect a stronger shape discrimination difference of the circle target from the distracting shape sequence than was the case for complex targets, and/or a more reliable recognition of the circle. The lack of an effect of delay duration on the proportion of false responses for simple trials might indicate that a prolonged maintenance period per se did not increase the cognitive demand in simple trials. In contrast, an increase of RTs and error rates with delay duration was observed for trials requiring the maintenance of complex shapes. Such a decrement in performance is a frequent finding in WM tasks with increasing memory load [21,38,39].

### WM maintenance

When contrasting maintenance periods between complex and simple conditions separately for each delay condition the resulting activation pattern was remarkably consistent across all four delay duration contrasts. This network comprising activation clusters primarily in frontal and parietal cortices is consistently found in WM maintenance processing during delayed match-to-sample and similar tasks [21,38,40], as well as n-back tasks [41]. Activation in the caudate nucleus is also frequently reported during WM processing [42,43]. Although we found a widespread activation pattern associated with the WM maintenance period the various brain activation areas might subserve different parts of WM processing in the present experimental design.

### MT^+^ modulation during WM maintenance with concurrent shape-monitoring

PSC values in MT^+^ during the maintenance period were found to be modulated by both the complexity of the memorized target and by the delay duration. In the monkey literature there is evidence for area MT being part of a network involved in WM processes [10,44]. In particular, the response to test stimuli in a delayed match-to-sample task was shown to be modulated by a previously shown sample stimulus, possibly due to an involvement of area MT during the comparison with the sample stimulus [44]. The task in the present study strongly requires the ongoing comparison of continuously changing shapes with the initially shown sample stimulus.

However, a major difference to other studies that have shown area MT engagement in memory processing [9,10,44] is the fact that the present task required the maintenance of a target shape and a continuous comparison with morphing probe shapes. Hence, there was no motion-related WM content. Indeed, there are some alternative explanations apart from pure differences in retention activity that might illustrate how task processing differences between complex and simple shape conditions might have contributed to the reported MT^+^ activation.

### Alternative explanations

Apart from memory retention, what kind of processing may underlie area MT^+^ activation during the maintenance period?

Previous studies have shown that neuronal responses in area MT^+^ can be modulated by attention in non-human primates [45,46] as well as in humans [47-50]. Moreover, the monkey area FST (the fundal surface of the superior temporal area) is located close to monkey area MT and possibly included in human area MT^+^. This region has been suggested to play a key role not only in motion processing but also in shape processing [51-53]. In addition, subregions of area MT^+^ have been reported to be engaged in object-selective processing [3,4]. Thus, the present task might have required a more specific pattern analysis when processing complex shapes containing more details like curvatures and bulges. Furthermore, different complex shapes might have been perceived more similar than complex shapes and the circle. Accordingly, the discrimination between different complex shapes during the maintenance period might have been more demanding than the discrimination between the complex shapes and the simple and well-known circle. Consequently, more attentional resources were captured to focus on the morphing process. These circumstances might have led to a higher attentional load and more demanding object-selective processing in MT^+^ during complex trials.

Another explanation for the MT^+^ modulation can be derived from the concept of object-based attention. Several studies have shown that attention can spread from the task-relevant feature of an attended object to other task-irrelevant features, leading to enhanced processing of multiple features of the attended object [54-56]. In the present continuous morphing task, participants had to observe the morphing shape during the delay and to recognize the target shape. Thus, attention might have spread from the relevant feature to another feature (more precisely, from shape to motion) leading to measurable attention effects in area MT^+^ in the complex conditions by posing higher attentional demands. Recent results corroborate this interpretation, as attention effects for the feature ‘color’ were found in human area MT^+^[57] and also in single cell recordings in area MT in macaque monkeys [58].

Finally, the ‘load theory’ [59] might provide a further explanation for the PSC modulation in area MT^+^. According to this theory more extensive processing of distractor stimuli is expected under higher WM demands. A study by De Fockert and colleagues [60] demonstrated higher activations in face processing areas in response to distractor faces in the higher WM-load condition, and larger susceptibility to distractor-related interference in this condition.

Conferred to the present study, the higher WM load associated with complex trials and increasing delay duration possibly also induced enhanced interference susceptibility. In this case, the morphing sequence with continuously changing shape outlines might have served as a distractor that interfered with WM maintenance during the delay period. Therefore, under higher WM load area MT^+^ could show increased activity due to its specific engagement in the processing of a distracting moving sequence.

In summary, apart from the initial interpretation of WM maintenance activity, the PSC pattern in area MT^+^ might also be explained either by higher attentional demands on object-selective processing mechanisms, by effects of object-based attention, or by enhanced processing demands due to the distracting morphing sequence.

All of these possible explanations go beyond the simple interpretation of MT^+^ as a visual area merely involved in motion processing.

The present data extend previous research as they show an increased involvement of area MT^+^ during the maintenance epoch of a WM task when complex stimuli are held in WM compared to the situation when a simple circle is maintained. Furthermore, these results were obtained with concurrent visual stimulation throughout the maintenance period. Previously, delay-spanning activity was found to be disrupted by distracting stimulation during the delay in posterior brain areas [15-17]. Our finding is particularly remarkable when considering that no motion-related information had to be maintained in WM in the present task. Indeed, this might challenge the view that brain regions showing activity in fMRI despite distracting stimulation or showing WM load dependent activity modulations are per se engaged in active maintenance. The idea that elevated activity per se does not necessarily imply active retention of stimulus information is in line with recent findings using a multi-voxel pattern analysis approach [61]. In this study, no stimulus-specific information could be detected in parietal or frontal regions showing increased and sustained delay activity. Potentially, a characteristic increase in activity with increasing WM demands (that was primarily thought to represent active maintenance mechanisms) might arise when brain regions are specifically involved in task processing, like area MT^+^ in the present task with continuously moving stimuli.

### Possible limitations of the present study and further research

From a methodological perspective, one may argue that the differences reported for the maintenance epochs of complex and simple control trials merely arise from differences in activity during the encoding epoch that should be more demanding for complex stimuli. In our view, this argument does not hold for two reasons: First, an assumed impact of encoding activity differences on the maintenance epoch would disappear with increasing retention length, leading to decreasing and then absent differences between simple and complex trials for longer delay durations. In contrast, PSC values in MT^+^ consistently showed pronounced differences for all delay durations. Second, an influence of encoding activity differences producing assumed maintenance differences should also lead to differences in the retrieval epoch, at least for short delay durations. In fact, there were no differences in MT^+^ PSC values for the retrieval epoch.

Another factor affecting the present data might be the difference in familiarity between the simple well-known circle and the different complex shapes that were at first unfamiliar but became more and more familiar during the experiment. Here, it is important to note that the familiarity of the visual stimulation was identical for the maintenance epochs of complex and simple conditions because only the ten complex shapes were presented during the delay and the circle never acted as a non-target morphing stimulus.

To further clarify the described cognitive load effect in area MT^+^ continuing experiments should be conducted. Using a pure shape discrimination task with no concurrent WM content but with a differing level of shape discrimination complexity will allow to investigate area MT^+^ activity exclusively associated with higher levels of shape discrimination.

Furthermore, modulating the attentional load of the distracting morphing sequence by using a parametrical manipulation of the distractor stimuli might also modify the MT^+^ response reported in the present study.

It might be also of interest to further differentiate the motion processing brain areas subsumed under the label ‘human MT^+^’ by the use of high-resolution fMRI and to investigate where the described cognitive load related MT^+^ activation originates from.

Nevertheless, those studies will always face a task-inherent issue, namely the problem to differentiate between attention-related and WM-related processes in a clear-cut fashion. Higher WM load might always be associated with higher attentional demands.

## Conclusions

Using a WM task variant with morphing stimuli during the delay and retrieval period where the only difference between conditions was the complexity of the memorized stimulus set we found activation in a large network of brain regions.

Signal changes during the maintenance period in motion-sensitive area MT^+^ were modulated by both target complexity and delay duration. PSC values were higher for complex compared to simple trials and increased with longer maintenance periods.

This study provides another piece of evidence that human area MT^+^ activation will be modulated by various endogenous factors involving higher cognitive processing mechanisms, e.g. the complexity of the memorized target shape even if there are no differences in physical stimulation.

## Abbreviations

Area MT^+^: Human homolog of medial temporal area and medial superior temporal cortex; PSC: Percent signal change; RTs: Reaction times; WM: Working memory.

## Competing interests

The authors declare that they have no competing interests.

## Authors’ contributions

All authors contributed to the conception and design of the study, the interpretation of the data, and critically revised the manuscript for important intellectual content. DG performed data acquisition and analysis and wrote the first draft of the manuscript. All authors read and approved the final manuscript and agreed to be accountable for all aspects of the work.
